# Evidence summary for pain management during retinopathy of prematurity screening

**DOI:** 10.3389/fped.2025.1682939

**Published:** 2026-01-15

**Authors:** Chunlan Tao, Jinghua Tang, Jing Tu, Yongli Li, Yuqin Chen

**Affiliations:** 1 Department of Neonatology, Affiliated Hospital of North Sichuan Medical College, Nanchong, Sichuan, China; 2 Nursing School, North Sichuan Medical College, Nanchong, Sichuan, China; 3 Department of Nursing, Affiliated Hospital of North Sichuan Medical College, Nanchong, Sichuan, China

**Keywords:** infant, premature, retinopathy of prematurity, mass screening, pain management, evidence–based practice

## Abstract

**Background:**

Retinopathy of prematurity (ROP) is one of the leading causes of childhood blindness. Routine ROP screening in high–risk preterm infants is a fundamental and effective measure to prevent ROP; however, this screening process could cause pain to infants. This study aims to summarize the best evidence for pain management during ROP screening and provide a reference for the clinical practice of medical staff.

**Methods:**

We systematically searched the literature on pain management during ROP screening, including clinical practice guidelines, evidence summaries, systematic reviews, meta–analyses, clinical decision support tools, and expert consensus statements. The search period was from inception to 31 October 2024. Four reviewers independently evaluated the quality of guidelines, and two reviewers independently assessed the quality of systematic reviews and expert consensus statements. Subsequently, evidence was extracted and graded.

**Results:**

Eighteen articles were included: six guidelines, three clinical decision support tools, six systematic reviews, and three expert consensus statements. 92 pieces of evidence were extracted and categorized into five dimensions: multidisciplinary pain management teams for ROP screening, pain assessment, non–pharmacological interventions, pharmacological interventions and pain documentation. Twenty–five evidence–based recommendations were finally formulated.

**Conclusion:**

The best evidence–based strategies for pain management during ROP screening in preterm infants provide actionable guidance for clinical practice. Medical staff should strengthen training in neonatal pain management and implement combined pharmacological and non–pharmacological interventions to alleviate procedural pain during ROP screening.

## 1 Introduction

Retinopathy of prematurity (ROP) is a fundus disease characterized by abnormal retinal vascular proliferation (1) and constitutes the primary cause of childhood visual impairment worldwide (2). Epidemiological data show that with the increasing proportion of advanced maternal age and the improvement of neonatal treatment capacity, the proportion of preterm births has risen significantly. Consequently, the incidence of ROP in China has gradually increased; relevant data indicate that its incidence in China is 15%–20% (3). ROP is a preventable and controllable ophthalmic disorder, and routine ROP screening for high–risk preterm infants is a fundamental and effective measure for its prevention and treatment (4).

However, multiple studies demonstrate that blepharoopeners, scleral compressors, and ophthalmoscopes induce moderate to severe pain in preterm infants (5–8). Importantly, a recent prospective cohort study demonstrated that a majority of neonates experience severe pain immediately after ROP screening (9). In addition, the Chinese Evidence–Based Guidelines for Neonatal Pain Management (2023) (10) further identifies ROP screening as both a source of acute pain and a common painful procedure in the Neonatal Intensive Care Unit (NICU). As neonates cannot verbally express discomfort, medical staff may overlook their pain perception and consequently fail to implement appropriate interventions during painful experiences. ROP screening is not a one–time procedure, as it may require repeated examinations based on clinical progression.

Repeated painful stimuli can induce a series of adverse effects in infants, including potential impacts on long–term neurodevelopmental and socioemotional outcomes (11). Although multiple countries have formulated guidelines for ROP and neonatal pain, there is a lack of systematic summaries of pain management measures during ROP screening. Therefore, this study systematically summarizes the best evidence for pain management during ROP screening to provide a reference for formulating and implementing relevant pain management strategies.

## 2 Method

### 2.1 Problem establishment

This study adopted the PI-POST framework (12). Population (P): Preterm infants (with a gestational age of <37 weeks) undergoing ROP screening. Intervention (I): Pain management during ROP screening, including pain assessment and intervention strategies. Professional (P): Neonatal medical staff and ophthalmologists. Outcome (O): The key components of a comprehensive pain management protocol for ROP screening. These included multidisciplinary pain management teams for ROP screening, pain assessment, non–pharmacological interventions, pharmacological interventions and pain documentation. Setting (S): NICU. Type of evidence (T): Evidence summaries, systematic reviews, meta–analyses, clinical decision support tools, and expert consensus statements. This evidence summary was registered with the Fudan University Center for Evidence–Based Nursing (Registration No: ES20245709).

### 2.2 Evidence retrieval

Following the “6S” evidence model (13), we systematically searched the following resources: Up To Date, BMJ, GIN (Guidelines International Network), NICE (National Institute for Health and Care Excellence), SIGN (Scottish Intercollegiate Guidelines Network), Medlive, NZGG (New Zealand Guidelines Group), APS (American Pain Society), BPS (British Pain Society), IASP (International Association for the Study of Pain), AAP (American Academy of Pediatrics), RNAO (Registered Nurses' Association of Ontario), JBI (Joanna Briggs Institute), The Cochrane Library, Embase, CINAHL, PubMed, Web of Science, CNKI (China National Knowledge Infrastructure), CBM (China Biology Medicine disc), Wanfang Data (Wanfang Data Knowledge Service Platform), and VIP (Weipu Information Chinese Scientific Journal Database), with a search cutoff date of October 31, 2024.

Both Medical Subject Headings (*MeSH*) terms and free–text keywords were used in the search; the search terms included “retinopathy of prematurity OR prematurity retinopathies” “mass screening OR fundus examination” “non–nutritive sucking OR touch” “pain OR pain score”. The detailed search strategy is provided in Supplementary File 1.

### 2.3 Inclusion and exclusion criteria of evidence

#### 2.3.1 Inclusion criteria

(1) Neonates were selected as the research subjects. (2) Pain management during ROP screening was the research content. (3) If guidelines have updated or revised versions, only the latest versions are included. (4) Type of literature: clinical practice guidelines, evidence summaries, systematic reviews, meta–analyses, clinical decision support tools, and expert consensus statements.

#### 2.3.2 Exclusion criteria

(1) Literature for which full texts cannot be obtained through multiple channels, and with only abstracts available. (2) Literature with non–preterm infants as the research subjects. (3) Literature with incomplete information that cannot provide sufficient evidence. (4) Literature in languages other than Chinese and English. (5) Literature with duplicate publications. (6) Literature with unclear quality evaluation results or failing to pass quality evaluation.

### 2.4 Quality evaluation of the literature

Recommendations in clinical decision support tools are typically based on high–quality evidence that has been recently published and peer–reviewed, and are classified as high–level evidence. Therefore, up to date clinical decision support tools can be directly included due to their high quality.
The guidelines were evaluated using the AGREE II, which comprises 6 domains, 23 items, and 2 overall assessment items (14). Each item is rated on a 1–7 scale, with 1 indicating strong disagreement and 7 indicating strong agreement. The standardized domain score is calculated as: (Actual score − Minimum score)/(Maximum score − Minimum score) × 100%. Recommendation levels were determined based on the comprehensive score of each domain: level A refers to a score of ≥60% in all six domains, which can be directly recommended without revision; level B requires achieving ≥30% in at least three domains, and <60% in one or more domains, which are recommended after targeted revisions; level C refers to a score of <30% in three or more domains, not recommended (15). This study adopted guidelines meeting Level A or B criteria.
Systematic reviews and meta–analyses were assessed using the AMSTAR II (16). Items 2, 4, 7, 9, 11, 13, and 15 are designated as critical items. A “high” quality rating requires all critical items to be satisfied, with none or only one non–critical item failing to meet the criteria. A “medium” quality rating is defined when more than one non–critical item fails to satisfy assessment criteria. A “low” quality rating is assigned when at least one critical item is unmet, irrespective of non–critical item compliance. A “very low” quality rating is assigned when more than one critical item is unmet, regardless of non–critical item compliance (17). This study included only literature with high and medium quality ratings.
Expert consensus statements adopt the expert consensus statements evaluation criteria of the JBI Center for Evidence–Based Health Care (2016) (18). Items with more than 2 evaluations as “no” are classified as low quality (19) and thus excluded.

The guidelines were evaluated by 4 researchers with training in evidence–based practice, while 2 researchers assessed the quality of systematic reviews and expert consensus statements. If discrepancies arose during evaluation, a third party (the graduate supervisor) would makes a final decision.

### 2.5 Evidence extraction and summary

Evidence Extraction: Evidence with the same theme and overlapping content can be integrated into a single piece of evidence; evidence with the same theme but complementary content can be combined into one complete piece of evidence in accordance with logical relationships; for conflicting content on the same theme, the principle of prioritizing high–quality, newly published authoritative literature shall be followed; independent evidence items shall retain their original wording. Two researchers independently extracted and integrated relevant evidence from the included literature. In addition, one evidence–based nursing expert and two neonatal experts from our hospital were invited to conduct a review (all three experts hold associate senior titles or above, and two of them are master supervisors).
Evidence Summary: The JBI Evidence Grading System was adopted for evaluation (20). According to different study design types, the evidence was categorized into Levels 1–5, with Level 1 representing the highest quality and Level 5 the lowest.

## 3 Result

### 3.1 Search results

**Figure 1:**
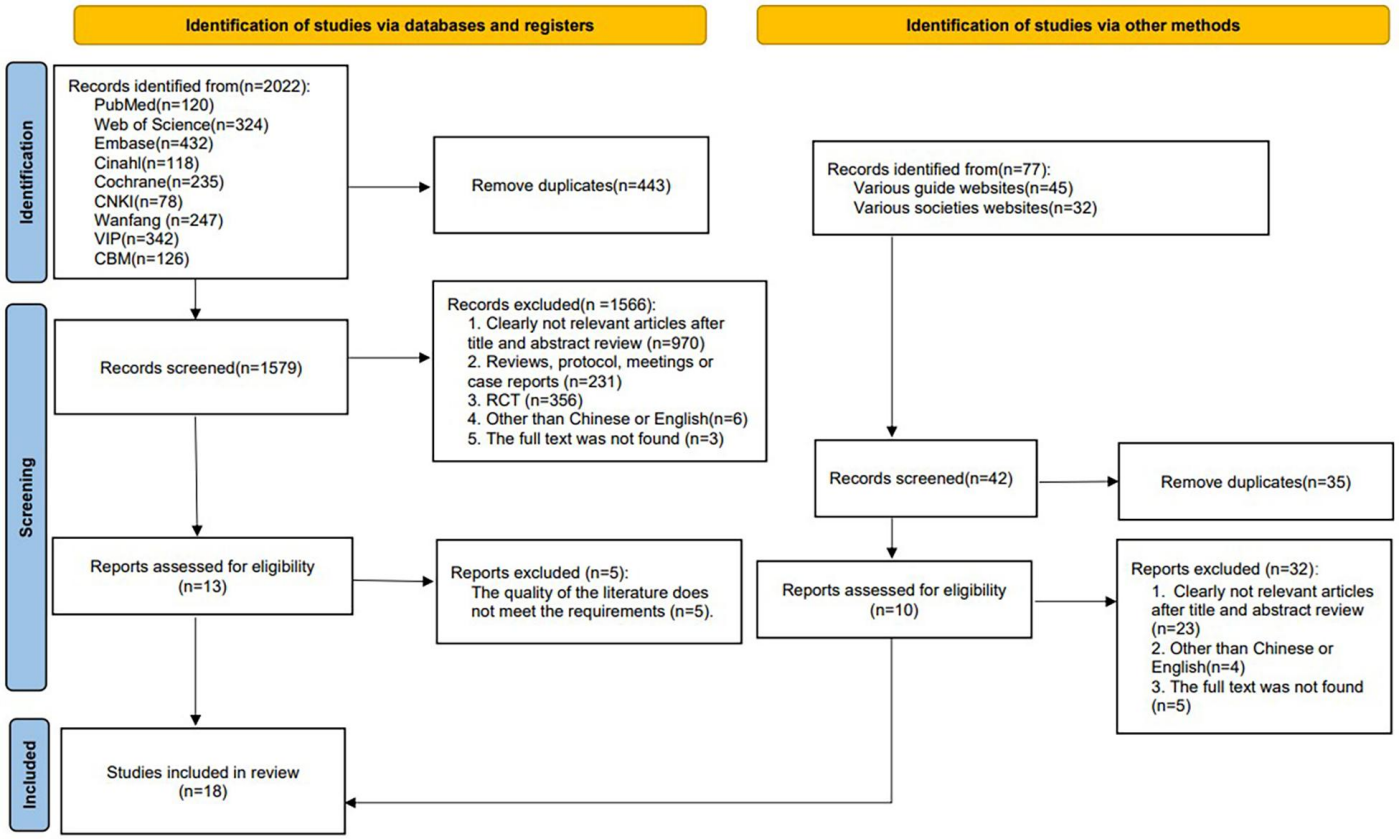
Literature screening flow diagram.

A total of 2,099 articles were retrieved through database searches. 1,621 articles were retained after removing duplicates. 1,598 irrelevant articles were excluded after screening titles and abstracts, leaving 23 articles. After reading the full text, 5 of these were excluded due to failure to meet quality evaluation criteria, and 18 articles were finally included. See the literature screening process in Figure 1.

### 3.2 Characteristics of included studies

Finally, 18 articles were included, with publication years ranging from 2011 to 2024. Among them, there were 6 guidelines (6/18, 33.3%), 3 clinical decision support tools (3/18, 16.7%), 6 systematic reviews and meta–analyses (6/18, 33.3%), and 3 expert consensus statements (3/18, 16.7%). In terms of region, 9 articles were from Asia (9/18, 50%), 4 from Europe (4/18, 22.2%), 4 from North America (4/18, 22.2%), and 1 from Africa (1/18, 5.56%), further details are presented in Supplementary File 2.

### 3.3 Literature quality evaluation results

#### 3.3.1 Clinical decision support tools

This study included three articles on clinical decision support tools from Up To Date (21–23), which were directly included.

#### 3.3.2 Clinical practice guidelines

Six guidelines were included (1, 10, 24–27), among which 3 were neonatal pain management guidelines (10, 24, 25), sourced from Wanfang, Medlive, and AAP; the other 3 were guidelines for ROP screening (1, 26, 27). These included 1 from the Wanfang Data (27) and 2 from Medlive (1, 26). The quality evaluation of the guidelines was performed by four researchers, and the results showed that 1 guideline was rated Level A (10) and 5 were rated Level B (1, 24–27), as shown in Supplementary File 3.

#### 3.3.3 Systematic reviews and meta–analyses

Six systematic reviews and meta–analyses were included (28–33), with four sourced from PubMed (28–30, 33), and two from Web of Science (31, 32). Their overall quality is acceptable; the detailed literature quality evaluation results are provided in Supplementary File 4.

#### 3.3.4 Expert consensus statements

Three expert consensus statements were included (2, 34, 35), with 2 sourced from Medlive (2, 34) and 1 from PubMed (35). Their overall quality is high, and the sources of the opinions are clearly marked and were thus included; the detailed literature quality evaluation results are provided in Supplementary File 5.

### 3.4 Best evidence summary

This study analyzed and summarized evidence pertaining to pain management during ROP screening, and finally extracted 25 recommendations across 5 aspects. Additionally, according to the JBI evidence grading and recommendation level system, the included evidence was evaluated, and the strength of recommendations was graded, further details are presented in Supplementary File 6.

## 4 Discussion

### 4.1 This evidence summary strictly adheres to an evidence–based methodology, with included literature subjected to rigorous screening to ensure evidence quality

The current study employed systematic literature searches for literature related to pain management during ROP screening. Through a rigorous literature screening process and quality assessment, evidence closely relevant to the topic was extracted and integrated. The quality assessment of the guidelines was performed by four individuals, while literature searching, evidence extraction, and evidence grading were independently conducted by two researchers. Additionally, the evidence summary underwent review by hospital–based evidence–based nursing specialists and neonatal care specialists to minimize the impact of the investigators' subjective biases and to establish a foundation for clinical pain management protocols.

### 4.2 Clinical implementation of the protocol developed in this evidence summary has guiding value for clinical practice

#### 4.2.1 Establish a multidisciplinary team for pain management during ROP screening

Evidence 1–2 suggests strictly adhering to relevant requirements for operators, enhancing pain management training for medical staff, and establishing a multidisciplinary team for pain management during ROP screening. Multidiscipline team for pain (PMDT) refers to a clinical treatment group composed of two or more disease–related experts. For pain management, it proposes scientific and reasonable diagnosis and treatment opinions that are proposed through regular and targeted meetings as part of a clinical treatment model (36). Lu et al. demonstrated that multidisciplinary teams involving parental participation are beneficial in relieving neonatal pain, promoting mother–infant attachment, alleviating parents' negative emotions, and enhancing caregiving ability and confidence (37). However, PMDT implementation in China is still in the initial stage. First, the total number of physicians engaged in ROP screening in China is insufficient, and there is a lack of standardized training. Second, medical staff have an insufficient understanding of pain management, which is mainly manifested in inadequate knowledge and a low assessment rate. In 2021, the Jiangsu Neonatology Medical Quality Control Center distributed questionnaires to NICUs' medical staff at its member units using a self–designed scale, and 957 valid questionnaires were collected. The average correct answer rate of medical staff regarding basic pain knowledge was approximately 30%, and the results indicated that most NICUs' medical staff were unable to correctly assess the degree of neonatal pain (38). In 2019, Shen et al. surveyed the current status of pain management practices in 66 medical institutions in China, with the results indicating that the pain assessment rate was only 39.02% (39). Finally, most NICUs adopt closed or semi–closed management, with insufficient human resources. In addition, parents have insufficient knowledge of pain management and low enthusiasm for participation (40). All these factors affect multidisciplinary pain management. Therefore, in the future, standardized training, particularly regarding pain–related knowledge, should be strengthened to establish of standardized training protocols. Evidence–based changes are carried out, Obstacles are identified, and management processes are standardized.

#### 4.2.2 Standardized pain assessment for ROP screening

Evidence 3–6 emphasizes mastery of appropriate clinical pain assessment tools and methods. Timely and accurate pain assessment is key to implementing pain management and plays a decisive role in developing effective interventions (10). Pain is an unpleasant emotional experience caused by actual or potential tissue damage, and it is a complex physiological and psychological activity (34). Studies have demonstrated that neonates experience an average of more than 5 pain stimuli per day during hospitalization, with the incidence of neonatal pain reaching 83.63% (6). Currently, no standards have been established for the selection of neonatal pain assessment scales (41). Over 40 scales have been applied in neonatal pain assessment. Among acute pain assessment tools, the NIPS (Neonatal Infant Pain Scale), PIPP (Premature Infant Pain Profile), DAN (Douleur Aiguë du Nouveau–né), and NFCS (Neonatal Facial Coding System) are primarily used both domestically and internationally. Among these, PIPP–R and N–PASS are more strongly recommended as assessment tools for pain during ROP screening. The PIPP–R comprises 7 items, including 3 behavioral indicators, 2 physiological indicators, and 2 baseline indicators. Its Internal consistency ranges from 0.94 to 0.98, indicating good applicability to preterm infants (42). N–PASS was sinicized by He et al. (43), which indicates that N–PASS has good reliability and validity, with internal consistency ranging from 0.84 to 0.89 when assessing acute neonatal pain. Compared with other scales, N–PASS has the advantages of a low bias risk, multi–dimensionality, ease of use, and rapidity. It is recommended by the American Academy of Pediatrics and the Neonatologist Branch of the Chinese Medical Doctor Association (10, 44, 45). Therefore, it is recommended to conduct high–quality neonatal pain assessments, adopt multi–dimensional assessment methods, select appropriate scales, and in non–emergency situations, combine multiple scales for neonatal pain assessment in non–emergency situations. This study recommends PIPP–R and N–PASS for pain assessment.

#### 4.2.3 Adopt a combination of non–pharmacological and pharmacological interventions to alleviate pain during ROP screening

Evidence 7–21 summarizes non–pharmacological interventions for alleviating ROP screening pain, including multisensory interventions, breastfeeding, non–nutritive sucking, oral sucrose, music therapy, and maternal voices.

Non–pharmacological interventions have the advantages of ease of use, safety, feasibility, easy learning, and easy implementation (46). The effect of a single intervention in alleviating neonatal pain is limited, and foreign researchers have demonstrated through meta–analyses and systematic reviews that combining multiple non–pharmacological interventions is superior to using a single intervention (29, 32). Among these, non–nutritive sucking (NNS) is the most widely used non–pharmacological analgesic method in neonatal wards. It exerts its effect by stimulating oral tactile receptors to increase the pain threshold and promoting the release of serotonin to induce analgesia (47). Additionally, sucking action can also achieve the effect of calming premature infants (48). Currently, multiple domestic and international guidelines recommend using NNS for pain management in neonates (10, 25). However, there is no consensus on the timing of NNS implementation, and the “Baby–Friendly Hospital Initiative” issued by the World Health Organization advocates minimizing pacifier use, primarily because early pacifier use may cause nipple confusion and improper disinfection or management can lead to infections (49). Therefore, researchers should select appropriate interventions based on actual clinical circumstances.

In addition, oral sweeteners are widely used in clinical practice. Neonates are administered sucrose water or glucose water via syringes, droppers, or pacifiers, triggering the release of endogenous opioids and activating taste receptors at the tip of the tongue to alleviate pain (50). Moreover, oral sweeteners have a sweet taste, stable properties, and a pleasant aftertaste, which can induce a pleasant sensation in neonates, effectively relieve negative emotions such as anxiety during painful procedures, and thereby alleviate their pain (51). However, inconsistencies exist among studies regarding the dosage, analgesic effect, and long–term outcomes of sweeteners, with no unified standards (10). In previous studies, Hou et al. administered glucose 2 min before the procedure (4), while Ren et al. administered glucose 1 min before the procedure for intervention (52). The impact of the administration timing on pain remains unknown.

Evidence 22–24 summarizes several studies indicating that topical anesthesia with proparacaine reduces pain scores. During ROP screening, proparacaine is commonly used as eye drops for topical anesthesia. However, despite routine ocular topical anesthesia prior to ROP screening, neonates still exhibit intense distressing behaviors. Additionally, certain pharmaceutical preparations may cause side effects such as hypotension and apnea in neonates (53).

Evidence 25 summarizes that structured electronic record forms can document the entire process of pain management, standardize pain management procedures, and effectively improve work efficiency.

This study summarizes the best evidence for pain during ROP screening, aiming to lay a theoretical foundation for developing a pain management plan, alleviate pain during ROP screening, and promote the continuous improvement of nursing quality.

## 5 Conclusion

This study adopts an evidence-based approach to extract and integrate the best evidence for pain management during ROP screening, providing a reference for clinical practice. The implementation of pain management during ROP screening can be achieved by establishing a multidisciplinary team for ROP screening pain management, strengthening pain management during ROP screening assessment, and combining non-pharmacological and pharmacological interventions. Future research will be conducted in two phases: first, implementing and validating the evidence-based pain management protocol within the specific context of China; second, exploring its adaptation and effectiveness in other international settings to promote global application.

## Data Availability

The original contributions presented in the study are included in the article/Supplementary Material, further inquiries can be directed to the corresponding author.
