# Supplementary material for: Evidence summary for pain management during retinopathy of prematurity screening
Source: Front Pediatr. 2026 Jan 15;13:1682939. doi: 10.3389/fped.2025.1682939 (PMC12852342; doi:10.3389/fped.2025.1682939)
Supplement: Supplementary file 1 [file Supplementaryfile1.docx]

**Supplementary**

Table of Contents:

[Supplementary 1: Search Strategy 2](#_Toc2896)

[Supplementary 2: Basic characteristics of the included literature 6](#_Toc9814)

[Supplementary 3: Quality Evaluation of Included Clinical practice guidelines 8](#_Toc32006)

[Supplementary 4: Quality Evaluation of Included Systematic reviews and Meta–analyses 9](#_Toc1179)

[Supplementary 5: Quality Evaluation of Included expert consensus statements 10](#_Toc543)

[Supplementary 6: Evidence Summary for Pain Management during ROP Screening 11](#_Toc26423)

# Supplementary 1: Search Strategy

***Search Strategy:***

| #1 | Search: infant, premature(MeSH Terms) |
| --- | --- |
| #2 | Search: infant, premature (Title/Abstract) OR prematur* (Title/Abstract) OR infant* (Title/Abstract) OR preterm infant* (Title/Abstract) OR neonat*(Title/Abstract) OR newborn*(Title/Abstract) |
| #3 | Search: (infant, premature(MeSH Terms)) OR (infant, premature (Title/Abstract) OR prematur* (Title/Abstract) OR infant* (Title/Abstract) OR preterm infant* (Title/Abstract) OR neonat*(Title/Abstract) OR newborn*(Title/Abstract)) |
| #4 | Search: Retinopathy of prematurity(MeSH Terms) |
| #5 | Search: Retinopathy of prematurity (Title/Abstract) OR ROP (Title/Abstract) OR Prematurity Retinopathies (Title/Abstract) OR Prematurity Retinopathy (Title/Abstract) |
| #6 | Search: (Retinopathy of prematurity(MeSH Terms)) OR (Retinopathy of prematurity (Title/Abstract) OR ROP (Title/Abstract) OR Prematurity Retinopathies (Title/Abstract) OR Prematurity Retinopathy (Title/Abstract)) |
| #7 | Search: Mass screening(MeSH Terms) |
| #8 | Search: Mass screening (Title/Abstract) OR screen*(Title/Abstract) OR examin*(Title/Abstract) OR diagnos*(Title/Abstract) OR detect*(Title/Abstract) OR monitor*(Title/Abstract) OR diagnostic techniques, ophthalmological (Title/Abstract) OR ophthalmic exam*(Title/Abstract) OR retinal exam*(Title/Abstract) OR Fundus screening (Title/Abstract) OR Fundus examination (Title/Abstract) OR Retinopathy screening |
| #9 | Search: (Mass screening(MeSH Terms)) OR (Mass screening (Title/Abstract) OR screen*(Title/Abstract) OR examin*(Title/Abstract) OR diagnos*(Title/Abstract) OR detect*(Title/Abstract) OR monitor*(Title/Abstract) OR diagnostic techniques, ophthalmological (Title/Abstract) OR ophthalmic exam*(Title/Abstract) OR retinal exam*(Title/Abstract) OR Fundus screening (Title/Abstract) OR Fundus examination (Title/Abstract) OR Retinopathy screening) |
| #10 | Search: Pain management(MeSH Terms) OR pain measurement(MeSH Terms) OR analgesia(MeSH Terms) OR analgesics(MeSH Terms) OR kangaroo-mother care method(MeSH Terms) OR Touch (MeSH Terms) OR Facilitated Tucking(MeSH Terms) OR sucrose(MeSH Terms) OR glucose(MeSH Terms) OR Breast Feeding(MeSH Terms) OR music therapy(MeSH Terms) |
| #11 | Search: Pain management(Title/Abstract) OR pain measurement(Title/Abstract) OR analgesia(Title/Abstract) OR analgesics(Title/Abstract) OR kangaroo-mother care method(Title/Abstract) OR Touch (Title/Abstract) OR Facilitated Tucking (Title/Abstract) OR sucrose (Title/Abstract) OR glucose (Title/Abstract) OR Breast Feeding (Title/Abstract) OR music therapy (Title/Abstract) OR Multi-sensory interventions(Title/Abstract) OR Multisensory(Title/Abstract) OR Multimodal(Title/Abstract) OR Non-nutritive sucking(Title/Abstract) OR pacifier (Title/Abstract) OR holding(Title/Abstract) OR Therapeutic Touch(Title/Abstract) OR skin-to-skin contact (Title/Abstract) OR SSC(Title/Abstract) OR holding (Title/Abstract) OR Maternal Holding (Title/Abstract) OR embracing breast milk sucking (Title/Abstract) OR EBMS (Title/Abstract) OR Swaddle (Title/Abstract) OR maternal voice (Title/Abstract)OR Traditional Chinese medicine horn sound (Title/Abstract)OR white noise (Title/Abstract) OR Breast milk smell(Title/Abstract) OR sucrose (Title/Abstract) OR BF(Title/Abstract) OR body position intervention(Title/Abstract) OR Curly position (Title/Abstract) OR nesting home(Title/Abstract) |
| #12 | Search: (Pain management(MeSH Terms) OR pain measurement(MeSH Terms) OR analgesia(MeSH Terms) OR analgesics(MeSH Terms) OR kangaroo-mother care method(MeSH Terms) OR Touch (MeSH Terms) OR Facilitated Tucking(MeSH Terms) OR sucrose(MeSH Terms) OR glucose(MeSH Terms) OR Breast Feeding(MeSH Terms) OR music therapy(MeSH Terms)) OR (Pain management(Title/Abstract) OR pain measurement(Title/Abstract) OR analgesia(Title/Abstract) OR analgesics(Title/Abstract) OR kangaroo-mother care method(Title/Abstract) OR Touch (Title/Abstract) OR Facilitated Tucking (Title/Abstract) OR sucrose (Title/Abstract) OR glucose (Title/Abstract) OR Breast Feeding (Title/Abstract) OR music therapy (Title/Abstract) OR Multi-sensory interventions(Title/Abstract) OR Multisensory(Title/Abstract) OR Multimodal(Title/Abstract) OR Non-nutritive sucking(Title/Abstract) OR pacifier (Title/Abstract) OR holding(Title/Abstract) OR Therapeutic Touch(Title/Abstract) OR skin-to-skin contact (Title/Abstract) OR SSC(Title/Abstract) OR holding (Title/Abstract) OR Maternal Holding (Title/Abstract) OR embracing breast milk sucking (Title/Abstract) OR EBMS (Title/Abstract) OR Swaddle (Title/Abstract) OR maternal voice (Title/Abstract)OR Traditional Chinese medicine horn sound (Title/Abstract)OR white noise (Title/Abstract) OR Breast milk smell(Title/Abstract) OR sucrose (Title/Abstract) OR BF(Title/Abstract) OR body position intervention(Title/Abstract) OR Curly position (Title/Abstract) OR nesting home(Title/Abstract)) |
| #13 | Search: Pain(MeSH Terms) |
| #14 | Search: Pain (Title/Abstract) OR Suffering (Title/Abstract) OR Physical (Title/Abstract) OR Physical Suffering (Title/Abstract) OR Physical Sufferings (Title/Abstract) OR Sufferings (Title/Abstract) OR Physical (Title/Abstract) OR Ache (Title/Abstract) OR Ache(Title/Abstract) OR manag*(Title/Abstract) OR relief(Title/Abstract) OR scor*(Title/Abstract) OR pain assessment(Title/Abstract) OR pain scale (Title/Abstract) OR pain score(Title/Abstract) |
| #15 | Search: (Pain(MeSH Terms)) OR (Pain (Title/Abstract) OR Suffering (Title/Abstract) OR Physical (Title/Abstract) OR Physical Suffering (Title/Abstract) OR Physical Sufferings (Title/Abstract) OR Sufferings (Title/Abstract) OR Physical (Title/Abstract) OR Ache (Title/Abstract) OR Ache(Title/Abstract) OR manag*(Title/Abstract) OR relief(Title/Abstract) OR scor*(Title/Abstract) OR pain assessment(Title/Abstract) OR pain scale (Title/Abstract) OR pain score(Title/Abstract)) |
| #16 | Search: (((((infant, premature(MeSH Terms)) OR (infant, premature (Title/Abstract) OR prematur* (Title/Abstract) OR infant* (Title/Abstract) OR preterm infant* (Title/Abstract) OR neonat*(Title/Abstract) OR newborn*(Title/Abstract))) AND ((Retinopathy of prematurity(MeSH Terms)) OR (Retinopathy of prematurity (Title/Abstract) OR ROP (Title/Abstract) OR Prematurity Retinopathies (Title/Abstract) OR Prematurity Retinopathy (Title/Abstract)))) AND ((Mass screening(MeSH Terms)) OR (Mass screening (Title/Abstract) OR screen*(Title/Abstract) OR examin*(Title/Abstract) OR diagnos*(Title/Abstract) OR detect*(Title/Abstract) OR monitor*(Title/Abstract) OR diagnostic techniques, ophthalmological (Title/Abstract) OR ophthalmic exam*(Title/Abstract) OR retinal exam*(Title/Abstract) OR Fundus screening (Title/Abstract) OR Fundus examination (Title/Abstract) OR Retinopathy screening))) AND ((Pain management(MeSH Terms) OR pain measurement(MeSH Terms) OR analgesia(MeSH Terms) OR analgesics(MeSH Terms) OR kangaroo-mother care method(MeSH Terms) OR Touch (MeSH Terms) OR Facilitated Tucking(MeSH Terms) OR sucrose(MeSH Terms) OR glucose(MeSH Terms) OR Breast Feeding(MeSH Terms) OR music therapy(MeSH Terms)) OR (Pain management(Title/Abstract) OR pain measurement(Title/Abstract) OR analgesia(Title/Abstract) OR analgesics(Title/Abstract) OR kangaroo-mother care method(Title/Abstract) OR Touch (Title/Abstract) OR Facilitated Tucking (Title/Abstract) OR sucrose (Title/Abstract) OR glucose (Title/Abstract) OR Breast Feeding (Title/Abstract) OR music therapy (Title/Abstract) OR Multi-sensory interventions(Title/Abstract) OR Multisensory(Title/Abstract) OR Multimodal(Title/Abstract) OR Non-nutritive sucking(Title/Abstract) OR pacifier (Title/Abstract) OR holding(Title/Abstract) OR Therapeutic Touch(Title/Abstract) OR skin-to-skin contact (Title/Abstract) OR SSC(Title/Abstract) OR holding (Title/Abstract) OR Maternal Holding (Title/Abstract) OR embracing breast milk sucking (Title/Abstract) OR EBMS (Title/Abstract) OR Swaddle (Title/Abstract) OR maternal voice (Title/Abstract)OR Traditional Chinese medicine horn sound (Title/Abstract)OR white noise (Title/Abstract) OR Breast milk smell(Title/Abstract) OR sucrose (Title/Abstract) OR BF(Title/Abstract) OR body position intervention(Title/Abstract) OR Curly position (Title/Abstract) OR nesting home(Title/Abstract)))) AND ((Pain(MeSH Terms)) OR (Pain (Title/Abstract) OR Suffering (Title/Abstract) OR Physical (Title/Abstract) OR Physical Suffering (Title/Abstract) OR Physical Sufferings (Title/Abstract) OR Sufferings (Title/Abstract) OR Physical (Title/Abstract) OR Ache (Title/Abstract) OR Ache(Title/Abstract) OR manag*(Title/Abstract) OR relief(Title/Abstract) OR scor*(Title/Abstract) OR pain assessment(Title/Abstract) OR pain scale (Title/Abstract) OR pain score(Title/Abstract))) |

# Supplementary 2: **Basic characteristics of the included literature**

| **Included literature** | **Source** | **Country/Region** | **Type of Literature** | **The Literature Theme** | **Year of Publication** |
| --- | --- | --- | --- | --- | --- |
| Bhatt A et al (21) | Up To Date | United States | Clinical decision support tools | Risk factors, classification and screening of retinopathy of prematurity | 2024 |
| Roué JM et al (22) | Up To Date | France | Clinical decision support tools | Neonatal pain assessment | 2024 |
| Jean-Michel Roué, MD, PhD et al (23) | Up To Date | France | Clinical decision support tools | Neonatal pain management and prevention | 2024 |
| Li T et al (24) | Wanfang Data | China | Clinical practice guidelines | Procedural Pain in Newborn | 2011 |
| Chinese Medical Doctor Association Neonatologist Branch (1) | Medlive | China | Clinical practice guidelines | Oxygen Therapy and Prevention of Retinopathy in Preterm Infants | 2013 |
| Guoming Z et al (26) | Medlive | China | Clinical practice guidelines | Screening for retinopathy of prematurity and neonates' eye disease | 2014 |
| Chinese Medical Association Ophthalmology Branch Fundus Disease Group (27) | Wanfang Data | China | Clinical practice guidelines | Retinopathy Screening Guidelines for Preterm Infants in China | 2014 |
| AAP et al (25) | AAP | United States | Clinical practice guidelines | Prevention and Management of Procedural Pain in the Neonate | 2016 |
| Qiao S et al (10) | Medlive | China | Clinical practice guidelines | Neonatal pain management in China | 2023 |
| Disher T et al (28) | PubMed | Canada | Meta–analyses | Pain-Relieving Interventions for Retinopathy of Prematurity | 2018 |
| Morgan ME et al (31) | Web of Science | United States | Systematic reviews | Neonatal Pain, Agitation, and Sedation Scale's use, reliability, and validity | 2020 |
| Thirunavukarasu, A J et al (33) | NGC | United Kingdom | Systematic reviews | Analgesia for retinopathy of prematurity screening | 2022 |
| Fajolu, I B et al (29) | PubMed | Nigeria | Systematic reviews | Non-pharmacological pain relief interventions in preterm neonates undergoing screening for retinopathy of prematurity | 2023 |
| Erçelik, Z E et al (30) | PubMed | Turkey | Systematic reviews and Meta–analyses | Nonpharmacological Applications During the Retinopathy of Prematurity Examination and Their Effects on Pain Control | 2022 |
| Qiaohong L et al (32) | Web of Science | China | Systematic reviews and Meta–analyses | Efficacy and safety of combined oral sucrose and nonnutritive sucking in pain management for infants | 2022 |
| Julia Harris et al (35) | PubMed | Netherlands | Expert consensus statements | Pain, sedation, withdrawal and delirium assessment in critically ill infants and children | 2016 |
| Daoman X et al (2) | Medlive | China | Expert consensus statements | Neonatal fundus screening | 2018 |
| Rui C et al (34) | Medlive | China | Expert consensus statements | Neonatal pain assessment and analgesia management | 2020 |

# Supplementary 3: **Quality Evaluation of Included Clinical practice guidelines**

| **Included literature** | **Percentage of Field Standardisation（%)** | | | | | | **≥60%** | **≥30%** | **Quality Grade** |
| --- | --- | --- | --- | --- | --- | --- | --- | --- | --- |
|  | **Scope and**  **Purpose** | **Stakeholder**  **Involvement** | **Rigour** | **Clarity** | **Applicability** | **Independence** |  |  |  |
| Li T et al (24) | 93.06 | 73.61 | 40.1 | 94.44 | 41.67 | 33.33 | 3 | 6 | B |
| Chinese Medical Doctor Association Neonatologist Branch (1) | 72.22 | 93.06 | 36.98 | 66.67 | 33.33 | 35.42 | 3 | 6 | B |
| Guoming Z et al (26) | 61.11 | 77.78 | 45.31 | 68.06 | 52.08 | 33.33 | 3 | 6 | B |
| Chinese Medical Association Ophthalmology Branch Fundus Disease Group (27) | 73.61 | 83.33 | 50 | 83.33 | 43.75 | 33.33 | 3 | 6 | B |
| AAP et al (25) | 88.89 | 77.78 | 34.38 | 90.28 | 35.42 | 47.92 | 3 | 6 | B |
| Qiao S et al (10) | 94.44 | 95.83 | 92.71 | 95.83 | 82.29 | 95.83 | 6 | 6 | A |

# Supplementary 4: Quality Evaluation of Included Systematic reviews and Meta–analyses

| **Included literature** | **①** | **②** | **③** | **④** | **⑤** | **⑥** | **⑦** | **⑧** | **⑨** | **⑩** | **⑪** | **⑫** | **⑬** | **⑭** | **⑮** | **⑯** |
| --- | --- | --- | --- | --- | --- | --- | --- | --- | --- | --- | --- | --- | --- | --- | --- | --- |
| Disher T et al (28) | Yes | Yes | Yes | Yes | Yes | Yes | Yes | Yes | Yes | No | Yes | Yes | Yes | Yes | Yes | Yes |
| Morgan ME et al (31) | Yes | Yes | Yes | Yes | Yes | Yes | Yes | Yes | Yes | No | Yes | Yes | Yes | 否 | Yes | Yes |
| Thirunavukarasu, A J et al (33) | Yes | Yes | Yes | Yes | Yes | Yes | Yes | Yes | Yes | No | Yes | Yes | Yes | Yes | Yes | Yes |
| Fajolu, I B et al( 29) | Yes | Yes | Yes | Yes | Yes | Yes | Yes | Yes | Yes | No | Yes | Yes | Yes | Yes | Yes | Yes |
| Erçelik, Z E et al (30) | Yes | Yes | Yes | Yes | Yes | Yes | Yes | Yes | Yes | No | Yes | Yes | Yes | Yes | Yes | Yes |
| Qiaohong Li et al (32) | Yes | Yes | Yes | Yes | Yes | Yes | Yes | Yes | No | No | Yes | Yes | Yes | Yes | Yes | Yes |

**Note**: ① Did the research questions and inclusion criteria for the review include the components of PICO? ② Did the report of the review contain an explicit statement that the review methods were established prior to the conduct of the review and did the report justify any significant deviations from the protocol? ③ Did the review authors explain their selection of the study designs for inclusion in the review? ④ Did the review authors use a comprehensive literature search strategy?⑤ Did the review authors perform study selection in duplicate? ⑥ Did the review authors perform data extraction in duplicate? ⑦ Did the review authors provide a list of excluded studies and justify the exclusions? ⑧ Did the review authors describe the included studies in adequate detail? ⑨ Did the review authors use a satisfactory technique for assessing the risk of bias (RoB) in individual studies that were included in the review? ⑩ Did the review authors report on the sources of funding for the studies included in the review? ⑪ If meta-analysis was performed, did the review authors use appropriate methods for statistical combination of results? ⑫ If meta-analysis was performed, did the review authors assess the potential impact of RoB in individual studies on the results of the meta-analysis or other evidence synthesis? ⑬ Did the review authors account for RoB in primary studies when interpreting/discussing the results of the review? ⑭ Did the review authors provide a satisfactory explanation for, and discussion of, any heterogeneity observed in the results of the review? ⑮ If they performed quantitative synthesis did the review authors carry out an adequate investigation of publication bias (small study bias) and discuss its likely impact on the results of the review? ⑯ Did the review authors report any potential sources of conflict of interest, including any funding they received for conducting the review?

# Supplementary 5: Quality Evaluation of Included expert consensus statements

| **Included literature** | **①** | **②** | **③** | **④** | **⑤** | **⑥** |
| --- | --- | --- | --- | --- | --- | --- |
| Julia Harris et al (35) | Yes | Yes | Yes | Yes | Yes | No |
| Daoman X et al (2) | Yes | Yes | Yes | Yes | Yes | No |
| Rui C et al (34) | Yes | Yes | Yes | Yes | Yes | No |

**Note:** ① Is the source of the opinion clearly stated? ② Does the source of opinion have standing in the field? ③ Are the interests of the relevant population the central focus? ④ Is the stated position the result of an analytic process, and is there logic in the opinion expressed? ⑤ Is there reference to the extant literature? ⑥ Is any incongruence with the literature identified?

# Supplementary 6: **Evidence Summary for Pain Management during ROP Screening**

| **Aspects** | **Description of Evidence** | **Evidence Level** |
| --- | --- | --- |
| Multidisciplinary pain management teams for ROP screening | 1. The ROP screening is performed by ophthalmologists specializing in neonatal diseases (1, 2, 21, 26). A multidisciplinary team for pain management of ROP screening is established, with core members including ophthalmologists, neonatologists, and neonatal nurses. | 5b |
|  | 1. Ensure that medical staff require standardized training in neonatal pain management, encompassing pain identification competencies. Correctly and effectively using clinical assessment tools to minimize inter-rater variability (22, 34). | 5b |
| Pain assessment | 1. A pain assessment tool appropriate for neonates should be selected (22, 25). Among these tools, N-PASS* (10, 31) and PIPP-R* (25, 35) are strongly recommended. In non-emergency scenarios, several scales can be combined for assessment (34). | 1a |
|  | 1. Neonatal pain assessment is routinely conducted (9, 21, 34), and standardized assessment before, during, and after the procedure is recommended for neonates undergoing planned pain-inducing procedures (10). | 5b |
|  | 1. Multimodal assessment of multiple indicators, incorporating physiology and behavior indicators, should be employed (25, 34). | 5b |
|  | 1. The assessment encompasses pain intensity, pain duration, and accompanying symptoms (10). | 5b |
| Non–pharmacological interventions | 1. Multimodal approaches tend to be more effective than single interventions, and combining non-pharmacological measures has a synergistic effect (23). | 2a |
|  | 1. NNS may be combined with oral sweeteners for combined intervention (10, 32, 34). | 2a |
|  | 1. Analgesic effects can be achieved through multisensory stimuli, including taste, smell, and touch (23, 28, 29). | 1a |
|  | 1. Practice nest care by placing newborns on a soft cushion, positioning them with flexed boundaries and allowing unrestricted movement (33). | 1b |
|  | 1. During screening, the newborn should be placed in a supine position, and an assistant should hold both sides of the cheeks with both hands and stabilize the head position (2). | 5b |
|  | 1. The newborn should be positioned in a curled posture with their limbs gently held in a flexed posture (10). | 2c |
|  | 1. Kangaroo care stimulates the ventral tactile and proprioceptive systems and attenuates neonatal pain responses (10, 23). | 2b |
|  | 1. The use of a pacifier to enhance neonatal sucking without the inhalation of breast milk or other sweeteners is the most commonly employed non-pharmacological analgesic method in neonatal units (10, 23, 25, 29). | 1b |
|  | 1. Touch therapy is implemented by scientifically and skillfully touching the newborn's skin and body, which transmits signals through skin receptors to the central nervous system, and sends excitatory vagus nerve signals to achieve the purpose of resisting pain and injury (23, 29) | 2b |
|  | 1. Swaddling should avoid overly tight swaddling as this may increase the risk of developmental dysplasia of the hip (2, 23). | 2c |
|  | 1. Administer orally either 0.2–0.5 ml/kg of 25% glucose solution or other sweetening agents 2 minutes before the operation (10, 23, 30, 33). | 1b |
|  | 1. Breastfeeding is recommended (10, 21, 23, 25), it is advised that the examination time should be separated from the feeding time (24, 34). | 2b |
|  | 1. Environmental noise in neonatal wards should be controlled, and environmental pain management measures such as music therapy should be adopted (10, 23, 24, 29, 34). Specifically, classical music is played through speakers, which are placed 30 cm from the head of preterm infants | 1d |
|  | 1. Recorded maternal heartbeats, mothers' speech, singing, or storytelling sounds, and sounds emitted by the mother in the neonatal ward were used to simulate the sound stimuli that newborns receive in utero (10). | 1c |
|  | 1. Olfactory stimulation using breast milk, vanilla, or lavender scents effectively reduces procedural pain in newborns (10). | 2c |
| Pharmacological interventions | 1. Surface anesthetics such as proparacaine should be used before the examination (10, 21, 23, 33, 34). | 1a |
|  | 1. Short-acting sedatives or opioids such as ketamine should be used as needed (23, 34). | 5b |
|  | 1. Intervention with local anesthetics combined with non-pharmacological interventions is recommended (28, 33). | 1a |
| Pain documentation | 1. A structured electronic record sheet is used to record the entire process of pain management: the date and time of assessment and intervention, pain assessment tools, pain assessment results (pain level, accompanying symptoms), intervention measures (non-pharmacological and pharmacological interventions), post-intervention pain scores, adverse reactions, and their management (10). | 5b |

**Note:** N–PASS(Neonatal Pain, Agitation and Sedation Scale), PIPP(Premature Infant Pain Profile)
